# Case of Popliteal Aneurism

**Published:** 1826-04

**Authors:** James M. Arnott

**Affiliations:** Surgeon.


THE LONDON

### Medical and Physical Journal.

No. 4 of VOL. LV.] APRIL, 1826. [No. 326.

For many fortunate discoveries in medicine, and for the detection of numerous errors, the world is indebted to the rapid circulation of Monthly Journals; and there never existed any work, to which the Faculty, in Europe and America, were under deeper obligations, than to the Medical and Physical Journal of London, now forming a long, but an invaluable, series.—RUSH.

### ORIGINAL COMMUNICATIONS,

### SELECT OBSERVATIONS, &c.

ART. I.—*Case of Popliteal Aneurism.* By JAMES M. ARNOTT, Esq. Surgeon.

THE following case is recorded, not from there having been any thing peculiar in the aneurism of the ham, or in the operation for its cure, but from the circumstances under which the patient's death subsequently took place, at a short distance of time after his recovery, and from the appearances presented on dissection of the body.

On the 20th of October last, I was requested to visit John Williams, æt. thirty-four, a brickmaker, on account of a swelling in the left ham, which impeded the use of the limb, and prevented him following his occupation. On examination, the hollow between the ham-strings was found occupied and distended by a large tumor, which felt firm but semi-elastic, yielded little to pressure, but pulsated freely; and the pulsations of which were stopped by compression of the femoral artery in the groin, but recurred immediately on this pressure being removed.

A swelling had been first perceived in this situation in the preceding month of June, on his leaving an hospital into which he had gone for rheumatism, and where pains in the left knee having been treated without success as rheumatic, he had been led, on his coming out, to consult a private practitioner, who discovered a swelling in the ham, and referred to it as the cause of the pains. Since then the man had continued at his work of brickmaking, the tumor gradually augmenting in size, and with increased inconvenience to the motions of the joint, until within a few days of my seeing him, when the severity of the pain, and the inability of extending and resting firmly on the limb, compelled him to give up work, and induced him to resort for advice and assistance to Mr. Barrack, of Gower-street, North, by whom I had been asked to see the case. He could assign no cause for his complaint. The bones of the leg had been fractured some years before.

On tracing the course of the large arteries of the body, no other aneurismal tumor could be discovered. The pulsations of the heart, carefully examined, communicated no other impression than that of health: like those of the artery at the wrist, they were regular and equable, of natural frequency and strength. The patient's health was good, and his constitution apparently sound. The nature of his complaint was therefore explained to him; and he was informed that it was only to be remedied by an operation, a favourable issue of which, from the circumstances of his case, might fairly be anticipated. He was further recommended, on account of his circumstances and inconvenient situation, to go into an hospital, but he objected to this; and, having satisfied myself that his habits were temperate, his disposition tranquil, and that his wife was capable of making a prudent and a careful nurse, I undertook to perform the operation for him at his own house.

Accordingly, on the 28th of October, it was performed in the usual manner; with this difference only, that, in consequence of the small size of the room, the operation was performed upon the patient as he lay in bed, the surgeon kneeling at the bed-side whilst executing it. The artery was tied in the upper third of the thigh, before it gets beneath the sartorius muscle. A single middle-sized thread of dentist's silk was used as a ligature, which was carried round the vessel with as little separation of it as possible from its surrounding connexions, and merely such as was effected by the passage of the aneurism needle. The pulsations in the tumor stopped immediately on tying the ligature, and did not recur. The subsequent progress of the case was favourable; the wound healed in a fortnight, except at the point where the ligature was situated, which came away on the 29th of November, the thirty-first day after the operation, and the wound then closed. The tumor in the ham had now diminished one-half in size, and the patient began to move about and make use of his limb.

Six weeks after this, and nearly twelve subsequent to the operation, Williams, who had been going about, but had not yet returned to his regular employment, died suddenly, under the following circumstances. He had been sitting in his own room with a friend, on the evening of the 18th of January, having had his share of a pot of beer; which, however, was all he had taken that day. His friend went out to fetch some tobacco; and, as he had only to go the distance of a few doors, and was not detained in the shop, the quantity he required being kept ready made up, he was not absent more than three minutes. On his return, he found Williams, whom he left sitting by the fire, lying dead within the fender, and the clothes over the left shoulder, which was within a few inches of the grate, burning. It was afterwards ascertained that the integuments of this part were but slightly scorched. On raising him up, he was found to be lifeless, and was pronounced by a surgeon, who saw him in a few minutes afterwards, to be quite dead. It would appear probable, from the position in which the body was found, that, as soon as his friend had left the room, Williams had got up to reach some coals, which were on the opposite side of the fire-place; and that, in this attempt, he had sunk down within the fender, in the position already described.

On opening the body, the pericardium was found to be distended with blood; separated, however, into coagulum and serum. On removing this, the heart itself was examined, which led to the discovery of a small aneurismal swelling of that portion of the aorta which is included within the pericardium, and where it is still covered by fat, continuous with that of the heart. This swelling was not very prominent, and was similar in size, and somewhat in shape, to that of half of a small walnut. A spot, the size of a split pea, on the surface of the swelling, was of a dark colour, owing to the thinness of the parietes at this place; and in the centre of this spot was a minute aperture, barely sufficient to admit the point of the blow-pipe, through which the blood had proceeded, which, filling the cavity of the pericardium, and oppressing the action of the heart, had produced the patient's immediate death.

The limb which had been the subject of operation was next examined, and the artery removed, from the groin to below what remained of the aneurismal tumor. The ligature had been thrown around the vessel an inch and a half below the origin of the profunda. Its situation was indicated by an indentation, and, on stretching the tube, the two portions of artery were seen to recede a little at this place from each other, leaving a narrow space between them, which was seen to be transparent, and where the continuity of the two was only kept up by means of cellular membrane. The obliterated portion of artery was not quite an inch in extent; that of the upper extremity of the vessel measuring five-eighths of an inch in length, that of the lower a little more than two-eighths. The ends of both were firmly united and consolidated. The plug in the upper portion of artery terminated superiorly precisely on a level with a small branch going off from the trunk of the vessel. Below the obliterated portion, the artery preserved its natural size, and was quite pervious all the way down to the aneurism, and at one place where it was opened was found to contain blood (liquid). Opposite the aneurism, the artery became again impervious, and for the extent of upwards of an inch. What remained of the aneurismal sac was of the size of a hen's egg. It formed an abrupt tumor on the vessel. The contents were solid, densely laminated coagula of blood; the section presenting lamellæ of uniform colour and consistence.

*New Burlington-street; February* 1826.

